# ChaMP-CMD: A Phenotype-Blinded, Randomized Controlled, Cross-Over Trial

**DOI:** 10.1161/CIRCULATIONAHA.123.066680

**Published:** 2023-10-31

**Authors:** Aish Sinha, Haseeb Rahman, Abdel Douiri, Ozan M. Demir, Kalpa De Silva, Brian Clapp, Ian Webb, Ankur Gulati, Pedro Pinho, Utkarsh Dutta, Howard Ellis, Ajay M. Shah, Amedeo Chiribiri, Michael Marber, Andrew J. Webb, Divaka Perera

**Affiliations:** 1 British Heart Foundation Centre of Excellence and National Institute for Health Research Biomedical Research Centre at the School of Cardiovascular Medicine and Sciences (A.S., H.R., O.M.D., U.D., H.E., A.M.S., A.C., M.M., A.J.W., D.P.), King’s College London, UK.; 2 Department of Medical Statistics, School of Life Course & Population Sciences (A.D.), King’s College London, UK.; 3 Guys’ and St. Thomas’ NHS Foundation Trust, London, UK (K.D.S., B.C., I.W., A.G., P.P., A.J.W., D.P.).; 4 King’s College Hospital NHS Foundation Trust, London. UK (I.W., A.M.S.).

**Keywords:** coronary circulation, microvascular angina

## Abstract

**BACKGROUND:**

Angina with nonobstructive coronary arteries is a common condition for which no effective treatment has been established. We hypothesized that the measurement of coronary flow reserve (CFR) allows identification of patients with angina with nonobstructive coronary arteries who would benefit from anti-ischemic therapy.

**METHODS:**

Patients with angina with nonobstructive coronary arteries underwent blinded invasive CFR measurement and were randomly assigned to receive 4 weeks of amlodipine or ranolazine. After a 1-week washout, they crossed over to the other drug for 4 weeks; final assessment was after the cessation of study medication for another 4 weeks. The primary outcome was change in treadmill exercise time, and the secondary outcome was change in Seattle Angina Questionnaire summary score in response to anti-ischemic therapy. Analysis was on a per protocol basis according to the following classification: coronary microvascular disease (CMD group) if CFR<2.5 and reference group if CFR≥2.5. The study protocol was registered before the first patient was enrolled (International Standard Randomised Controlled Trial Number: ISRCTN94728379).

**RESULTS:**

Eighty-seven patients (61±8 years of age; 62% women) underwent random assignment (57 CMD group and 30 reference group). Baseline exercise time and Seattle Angina Questionnaire summary scores were similar between groups. The CMD group had a greater increment (delta) in exercise time than the reference group in response to both amlodipine (difference in delta, 82 s [95% CI, 37–126 s]; *P*<0.001) and ranolazine (difference in delta, 68 s [95% CI, 21–115 s]; *P*=0.005). The CMD group reported a greater increment (delta) in Seattle Angina Questionnaire summary score than the reference group in response to ranolazine (difference in delta, 7 points [95% CI, 0–15]; *P*=0.048), but not to amlodipine (difference in delta, 2 points [95% CI, –5 to 8]; *P*=0.549).

**CONCLUSIONS:**

Among phenotypically similar patients with angina with nonobstructive coronary arteries, only those with an impaired CFR derive benefit from anti-ischemic therapy. These findings support measurement of CFR to diagnose and guide management of this otherwise heterogeneous patient group.

**Clinical Perspective**

**What Is New?**

- In a phenotypically homogeneous patient cohort, invasively characterized coronary flow reserve (CFR) identifies those who may respond to anti-ischemic therapy.
- The optimal threshold of CFR that identifies responders to therapy is the same as the one that is used to diagnose coronary microvascular disease.

**What Are the Clinical Implications?**

- CFR measurement should be considered in patients with limiting angina and nonobstructive coronary arteries to identify those who will derive benefit from anti-ischemic therapy.
- Patients with an abnormal CFR responded to therapy, whereas those with a normal CFR did not, suggesting that physiology-stratified therapy may be preferable to treating undifferentiated patients with angina and nonobstructive coronary arteries with empirical therapy.
- Among patients with an impaired CFR, measurement of minimal microvascular resistance may enable tailored, pathobiology-directed, therapy.

Angina with nonobstructive coronary arteries (ANOCA) is a common clinical condition,^1^ although this umbrella term covers several distinct pathophysiological entities. These include coronary microvascular disease (CMD), defined as an inability of the coronary vasculature to increase coronary blood flow in response to heightened myocardial oxygen demand despite the absence of epicardial coronary artery disease.^2^ CMD leads to impaired quality of life^3^ and a heightened risk of adverse cardiovascular outcomes.^4^ A diagnosis of CMD is traditionally made by invasive assessment in the cardiac catheter laboratory^5^ to delineate the hallmark of CMD, a diminished coronary flow reserve (CFR), representing the ratio of maximal achievable flow to resting flow. CFR<2.5 is associated with impaired coronary perfusion efficiency during exercise and myocardial ischemia on noninvasive assessment.^2^ It has recently been demonstrated that CMD itself may be a heterogeneous condition comprising 2 distinct endotypes termed functional and structural CMD,^6^ distinguished by elevated minimal microvascular resistance in the latter. Although the 2 endotypes are phenotypically similar, the underlying pathobiology is distinct.^2,6^

Although the CorMicA study (Coronary Microvascular Angina) has previously demonstrated the value of coronary physiology-stratified therapy in enhancing angina-specific quality of life^7^ in patients with ANOCA (including patients with CMD, vasospastic angina, and noncardiac chest pain), uptake by physicians has been low; this is partly because of skepticism about the mechanistic link between coronary physiology parameters and response to therapy. Furthermore, it is not known whether physiology-stratified therapy leads to an improvement in exercise capacity, which is the reference standard for efficacy of anti-ischemic therapies.

The ChaMP-CMD trial (Characterising Mechanisms in Patients with Coronary Microvascular Disease to Stratify Therapy) aims to assess whether a diminished CFR relates to the effects of anti-ischemic therapy on exercise time in patients with ANOCA and, if it does, whether further endotyping, on the basis of minimal microvascular resistance, allows even more granular stratification of therapy.

## METHODS

### Study Design and Participants

ChaMP-CMD is a phenotype-blinded randomized crossover trial performed at a tertiary referral cardiac center in London, UK. This study was approved by the National Health Service Research Ethics Committee (reference 20/LO/1294), funded by the UK Medical Research Council, and overseen by a trial steering committee. Written informed consent was obtained from all patients before their enrollment. The study protocol was registered before the first patient was enrolled (International Standard Randomised Controlled Trial Number: ISRCTN94728379).

We recruited patients with predictable exertional chest tightness, preserved left ventricular ejection fraction (>50%), and unobstructed coronary arteries (fractional flow reserve >0.80). Exclusion criteria were intolerance to adenosine, advanced chronic kidney disease (estimated glomerular filtration rate <30 mL·min^–1^·m^–2^), significant valvular heart disease, history of acute coronary syndrome, previous revascularization, cardiomyopathy, atypical/noncardiac chest pain, low symptom burden, confirmed vasospastic angina (necessitating commencement of calcium channel antagonists), exercise incapacity due to noncardiac causes, known contraindications to ranolazine or amlodipine, patients who were unable to exercise on a treadmill or those who could exercise for >540 s in the absence of any revealed cardiac symptoms on the baseline exercise test.

### Randomization, Masking, and Crossover

Patients, researchers, and clinical staff conducting exercise tests were blinded to the patients’ invasive physiological characteristics (phenotype-blinding). Exercise-testing staff were also blinded to the study medication. All eligible study patients were randomly assigned to receive either amlodipine or ranolazine (in a 1:1 allocation ratio) using an automated randomization tool; no stratification or blocks were used. Patients who were already taking a calcium channel antagonist or ranolazine were requested to withhold them from 4 weeks before their baseline visit until the end of the study.

The initial doses were either amlodipine 5 mg once a day or ranolazine 375 mg twice a day, with an increase to amlodipine 10 mg once a day and ranolazine 500 mg or 750 mg twice a day after 1 to 2 weeks. If patients did not tolerate amlodipine 5 mg, then the dose was reduced to 2.5 mg. Four weeks after randomization, patients returned for the second exercise treadmill test (ETT), after which a 1-week drug-washout period was applied (a duration >5 half-lives of either medication, to minimize potential carryover effects^8^). They then crossed over to the second drug arm in the sequence of amlodipine-ranolazine or ranolazine-amlodipine, and after 4 weeks of treatment, returned for the third ETT. Study medications were discontinued at this point and a subgroup returned for a fourth ETT, 4 weeks later, to exclude training effects. Patients were encouraged to continue their regular (nonstudy) medications throughout the study period. Treatment adherence was assessed using pill count at each visit and patients with <80% adherence to study medications, or those who were not able to attend their second or third visit within 1 week of the scheduled date, were excluded from that aspect of the per protocol analysis but included in the intention-to-treat analyses. Ambulant day-to-day activity was monitored by capturing 4-week step counts from patients’ smartphone application.

**Figure 1.**
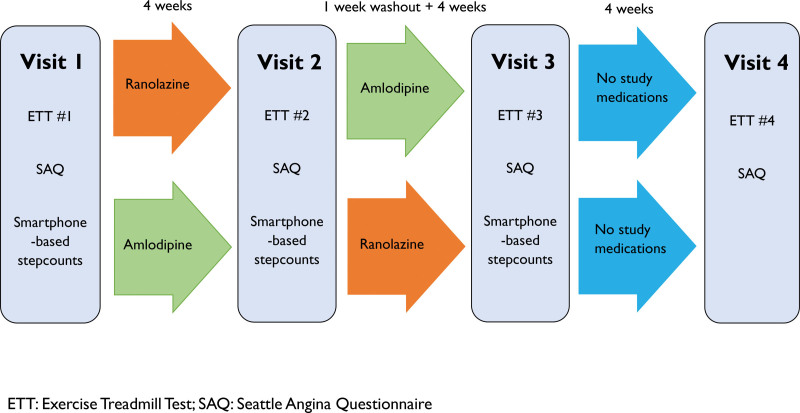
Study flow.

After the fourth ETT (or third for those who did not undertake the fourth ETT), study participation was complete, and patients and physicians were unblinded to the patients’ phenotype. Figure 1 illustrates the study flow.

### Procedures

#### Coronary Angiography

Coronary angiography was performed through the radial artery, with 1 mg intravenous midazolam, 70 U/kg intra-arterial unfractionated heparin, and 400 to 600 μg intracoronary isosorbide dinitrate administered at the start of the procedure. Quantitative coronary angiography was used to determine the diameter of stenosis. A calibrated 0.014-inch dual sensor–tipped intracoronary guidewire (ComboWire, Philips Volcano, Rancho Cordova, CA) was used for measurement of distal coronary pressure (P_d_) and average peak flow velocity. Aortic pressure (P_a_) was measured by the fluid-filled guide catheter. Hyperemia was induced with intravenous adenosine, administered through the brachial vein at 140 μg·kg^–1^·min^–1^. Signals were sampled at 200 Hz, with data exported into a custom-made study manager program (Academic Medical Centre, University of Amsterdam, Amsterdam, Netherlands) and analyzed on custom-made software, Cardiac Waves (King’s College London, London, UK). CFR was calculated as hyperemic average peak flow velocity/resting average peak flow velocity, both measured at steady state. Patients were classified off-line into reference (CFR≥2.5) and CMD (CFR<2.5) groups. Hyperemic (minimal) microvascular resistance was calculated as P_d_/average peak flow velocity and the CMD endotype defined as previously described^6^: functional CMD as CFR<2.5 and hyperemic (minimal) microvascular resistance <2.5 mm Hg·cm^–1^·s^–1^; structural CMD as CFR<2.5 and hyperemic (minimal) microvascular resistance ≥2.5 mm Hg·cm^–1^·s^–1^.

#### Exercise Treadmill Test

The ETT was performed with a Marquette Case 8000 system (GE Medical Systems, Milwaukee, WI) according to the American College of Cardiology and American Heart Association practice guidelines using a standard Bruce protocol^9^ and terminated at the patient’s request. We aimed to perform the first ETT within 4 weeks of patients’ coronary physiology characterization. Patients were required to withhold glyceryl trinitrate 60 minutes before exercise testing. Twelve-lead ECG, heart rate, and blood pressure were recorded at regular intervals before, during, and after the ETT. All ETTs were supervised by physiologists who were blinded to the patients’ coronary physiology data. Exercise time was defined as the time from the start of the exercise protocol to exercise cessation.

#### Seattle Angina Questionnaire

At each visit, patients completed the Seattle Angina Questionnaire (SAQ), which is a validated angina-specific quality-of-life assessment.^10^ The SAQ comprises 5 components, namely physical limitation, angina stability, angina frequency, treatment satisfaction, and quality of life; these are then incorporated into the summary score, which is sensitive to changes in response to therapy.^10^

### Outcomes

The primary outcome measure was the change in exercise time in response to anti-ischemic therapy. The secondary outcome measure was the change in SAQ summary score in response to anti-ischemic therapy.

### Hypotheses and Statistical Considerations

Our primary hypothesis was that patients with CMD will have a greater improvement in their exercise capacity in response to anti-ischemic therapy compared with patients with a normal CFR. Assuming a 2:1 distribution (as reported in the literature for patients with a high pretest probability of coronary vascular dysfunction), 49 patients with CMD and 25 with a normal CFR will provide 80% power (α=0.05) to detect a 60-s difference in exercise time between the groups (assumed SD, 85 s). On the basis of previous mechanistic work, and as discussed later, our second (hierarchical) hypothesis was that patients with *functional CMD* will have a greater improvement in their exercise capacity in response to ranolazine compared with amlodipine, whereas patients with *structural CMD* will have a greater improvement in their exercise capacity in response to amlodipine compared with ranolazine.^6^ Assuming a 1:1 distribution of endotypes, the sample size required to detect a difference of 60 s (SD, 85 s) in response to amlodipine and ranolazine, respectively, at 80% power and 5% significance is 18 patients of each endotype (for a within-group comparison). Given the hierarchical hypothesis testing, no adjustment of the significance level was applied (α=0.05). A statistical analysis plan was finalized before data lock and unblinded analysis of data.

Normality of data was assessed using the Kolmogorov-Smirnov test. Normally distributed continuous data are presented as mean±SD, unless specified otherwise, and compared using the independent sample’s Student *t* test. Nonnormally distributed data are presented as median (interquartile range) and compared using the Mann-Whitney test (unpaired analyses) or the Wilcoxon matched-pairs signed rank test (paired analyses). Categorical variables are presented as n (%) and compared using the χ^2^ test. Continuous end points were compared with the 2-sample t test of the difference between groups (CMD versus reference groups) or within groups (within structural CMD and within functional CMD); unpaired t tests were used for the between-group comparisons and paired t tests for the within-group comparisons. The findings are reported as the difference in mean change between (or within) study groups with 95% CIs and *P* values. Binary logistic regression was performed to identify predictors of ≥60 s increment in exercise time in response to anti-ischemic therapy (ranolazine or amlodipine), using univariable and multivariable analysis and reported as odds ratio (95% CI). Gain-or-loss of function was assessed using the repeated-measures ANOVA analysis (mixed model, assuming missing values are missing at random). Sphericity was not assumed, and the Geisser-Greenhouse correction was applied to the analyses. The Youden index in receiver operating characteristic curves was used to identify the optimal CFR threshold that predicted ≥60 s increment in exercise time in response to anti-ischemic therapy. All randomly assigned patients were included in these analyses. The accuracy of CFR thresholds in predicting ≥60 s increment in exercise time in response to anti-ischemic therapy was calculated as [(true positives + true negatives) ÷ (true positives + true negatives + false positives + false negatives)]×100; the accuracy of different CFR thresholds was compared using the McNemar test. Data were analyzed on a per protocol basis as stipulated in the Statistical Analysis Plan. Per protocol analysis was favored over intention-to-treat analysis because this study addressed the efficacy of therapy as opposed to a treatment strategy. Intention-to-treat analyses are also reported in the Supplemental Material. All analyses were performed using SPSS Statistics 27 (IBM) and GraphPad Prism software version 9.0 (GraphPad software, San Diego, CA). We deemed a *P* value <0.05 to be significant.

A summary of the protocol has been submitted with this article and is available at (https://doi.org/10.1186/ISRCTN94728379). The study was registered with the National Institute for Health Research UK Clinical Research Network portfolio database (Central Portfolio Management System identifier: 47795) and International Standard Randomised Controlled Trial Number (identifier: ISRCTN94728379).

## RESULTS

**Figure 2.**
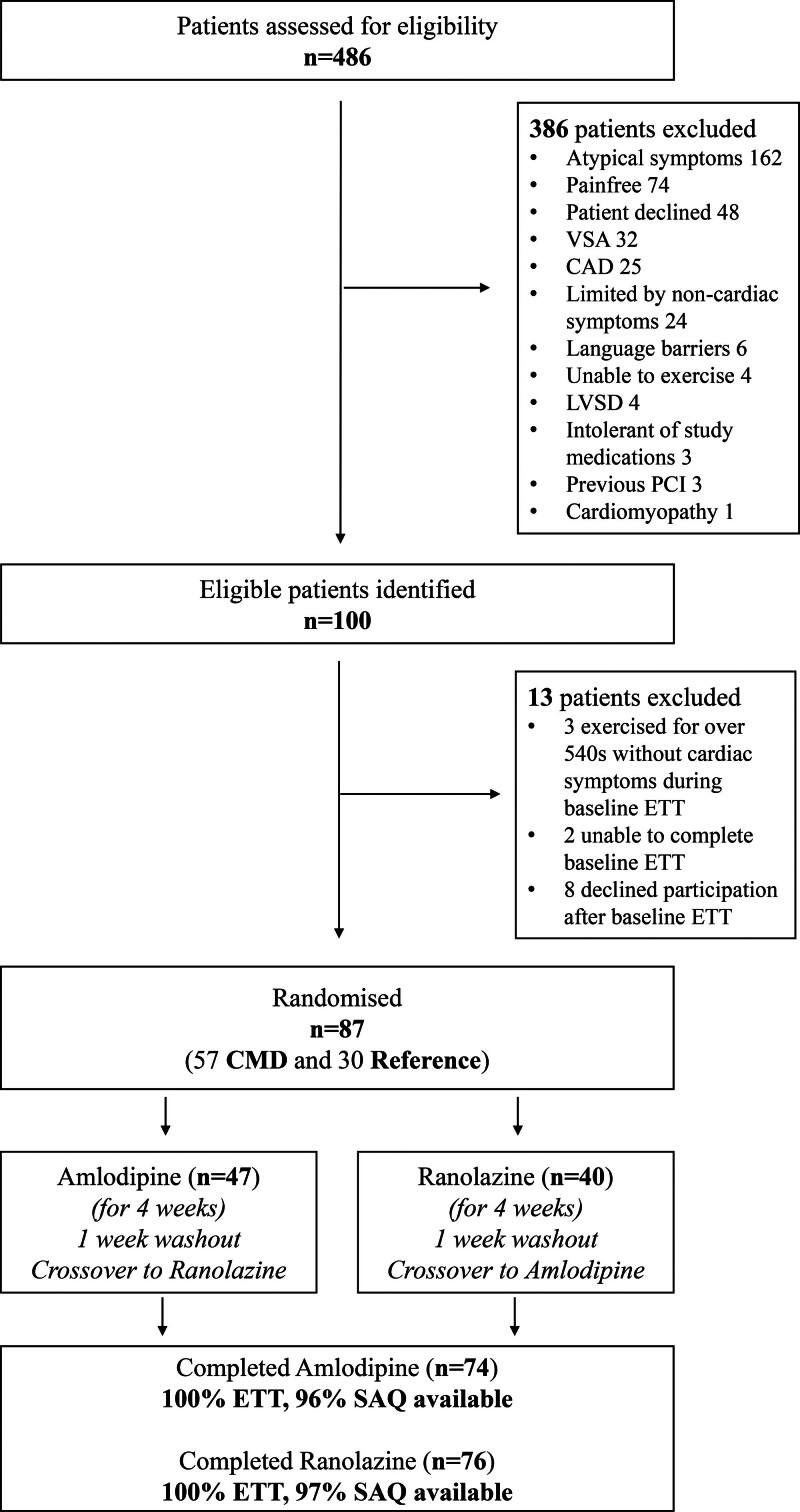
**Patient screening and recruitment numbers.** CAD indicates coronary artery disease; CMD, coronary microvascular disease; ETT, exercise treadmill test; LVSD, left ventricular systolic dysfunction; PCI, percutaneous coronary intervention; SAQ, Seattle Angina Questionnaire; and VSA: vasospastic angina.

Between December 17, 2020, and August 2 2023, 486 patients with angina and nonobstructive coronary arteries were screened and 100 eligible patients identified, of which 87 were randomly assigned (Figure 2). By blinded classification, 57 patients had impaired CFR (CMD group) and 30 patients had normal CFR (reference group). Of those with CMD, 22 patients had the *structural* endotype and 35 had the *functional* endotype. The ETT took place 25 (14–41) days after the coronary physiology assessment. Patients in the CMD and reference groups were well matched for baseline demographics; the CMD group had a higher prevalence of hypertension (Table 1). There were no differences in the epicardial coronary anatomy and physiology metrics (mean fractional flow reserve >0.90 in both groups), baseline exercise time (315±143 versus 317±128 s, *P*=0.954) or SAQ summary score (49±19 versus 51±19, *P*=0.651) between the CMD and reference groups (Table 2). Exercise time and SAQ summary score at baseline were moderately correlated (*r*=0.39, *r*^2^=0.15, *P*=0.003). Thirteen patients did not tolerate amlodipine (<80% adherence) or attend their ETT at the scheduled date while taking amlodipine, whereas 11 patients did not tolerate ranolazine or attend their ETT at the scheduled date while taking ranolazine. Patients were taking the following medication at the time of the second and third ETT: amlodipine 2.5 mg once a day (4%), amlodipine 5 mg once a day (61%), or amlodipine 10 mg once a day (35%); ranolazine 375 mg twice a day (47%), ranolazine 500 mg twice a day (11%), or ranolazine 750 mg twice a day (42%).

**Table 1.**
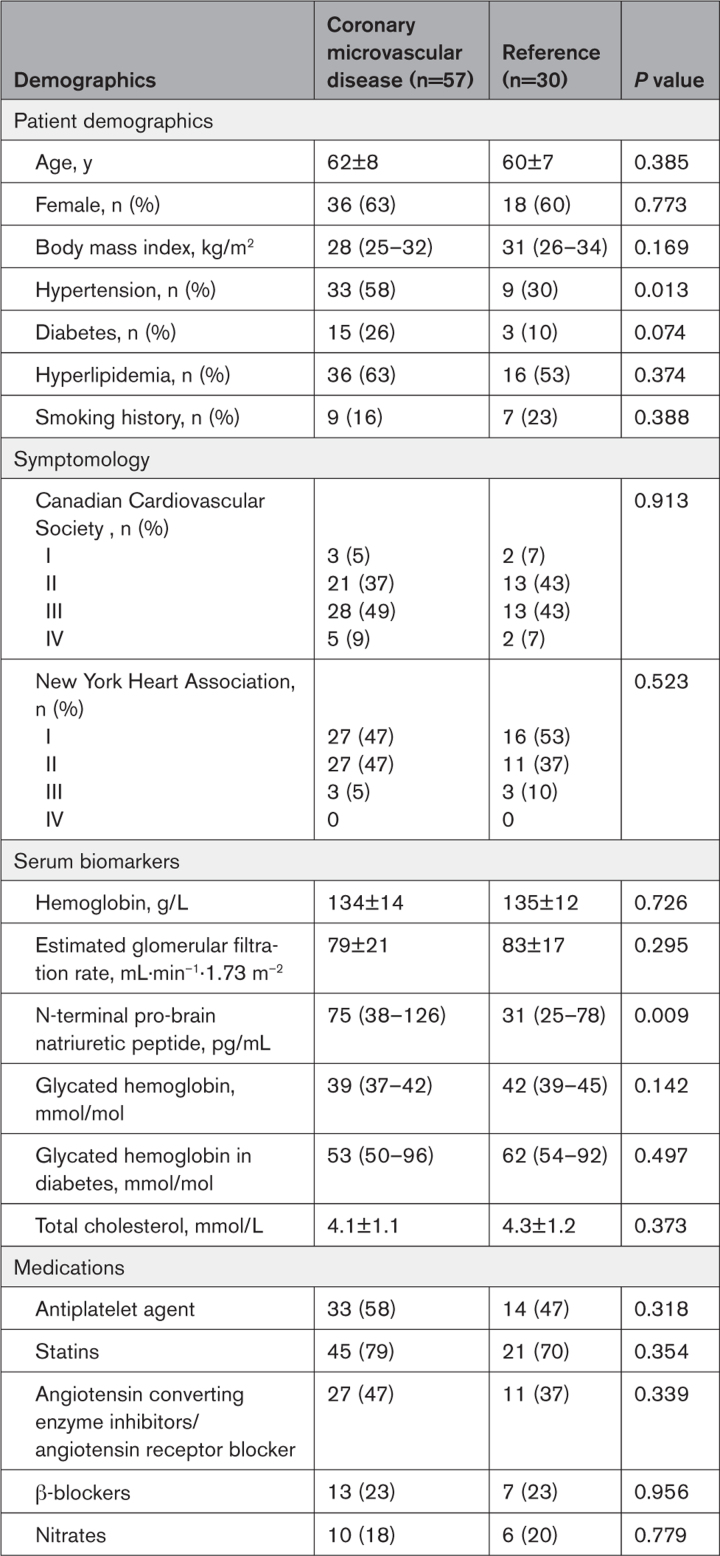
Baseline Demographics

**Table 2.**
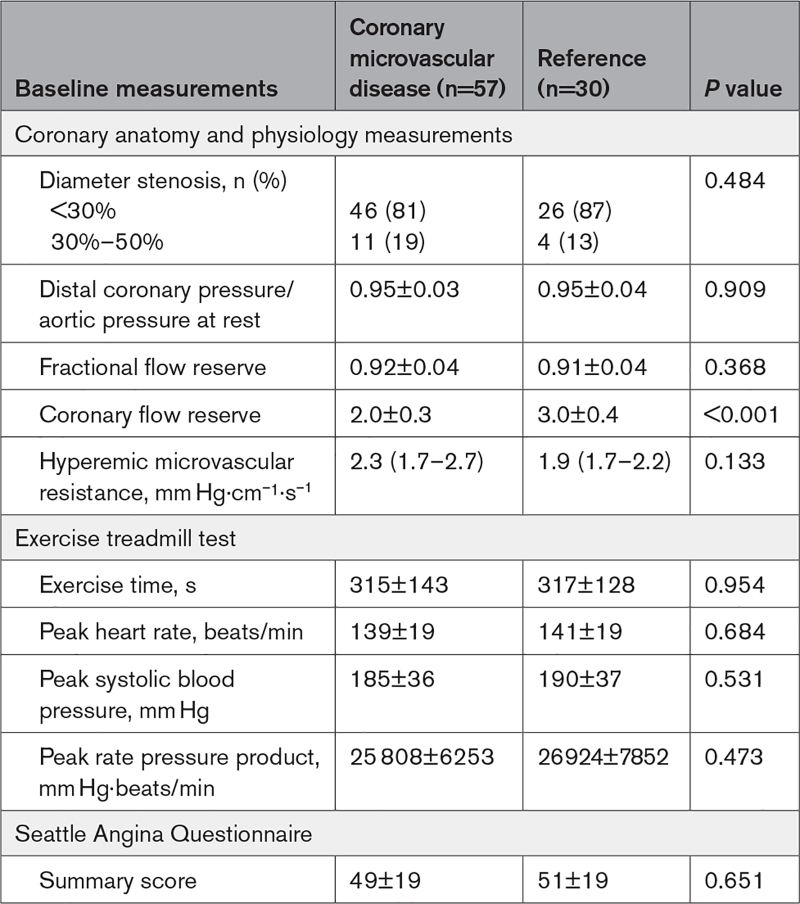
Coronary Physiology, Exercise Test, and Seattle Angina Questionnaire Measurements at Baseline.

**Figure 3.**
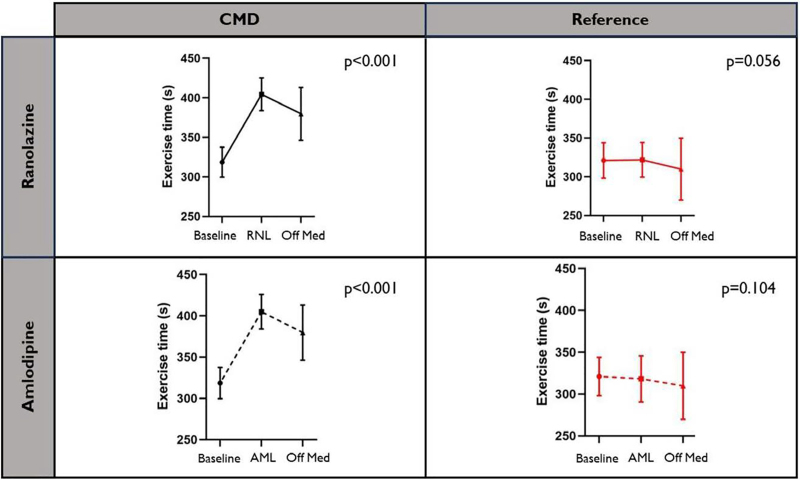
**Change in exercise time between baseline, with anti-ischemic medication and without anti-ischemic medication.** Data are presented as mean±SEM; *P* values are for repeated-measures ANOVA. AML indicates amlodipine; CMD, coronary microvascular disease; Off Med, after cessation of study medications; and RNL, ranolazine.

Patients with CMD had a greater increment in exercise time compared with the reference group with both amlodipine (mean difference in delta, 82 s [95% CI, 37–126 s], *P*<0.001) and ranolazine (mean difference in delta, 68 s [95% CI, 21–115 s], *P*=0.005; Table 3 and Table S1). In patients with a normal CFR, there was no change in exercise time (compared with baseline) in response to either anti-ischemic agent (amlodipine: mean change 9 s [95% CI, –20 to 38 s], p=0.526; ranolazine: mean change 11 s [95% CI, –20 to 42 s], *P*=0.469). The gain in exercise time observed in the CMD group was lost after the cessation of each anti-ischemic medication, as assessed on the 4th ETT (off study medication; *P*<0.001 for both amlodipine and ranolazine by repeated-measures ANOVA). In the reference group, exercise time remained unchanged, on or off the study medication (Figure 3). Additional ETT-related parameters are reported in Table S2 and Figures S1 and S2.

**Table 3.**
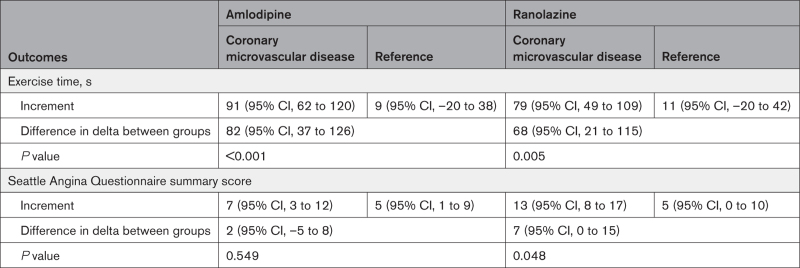
Primary (Difference in Delta in Exercise Time) and Secondary (Difference in Delta in Seattle Angina Questionnaire Summary Score) Outcomes

**Figure 4.**
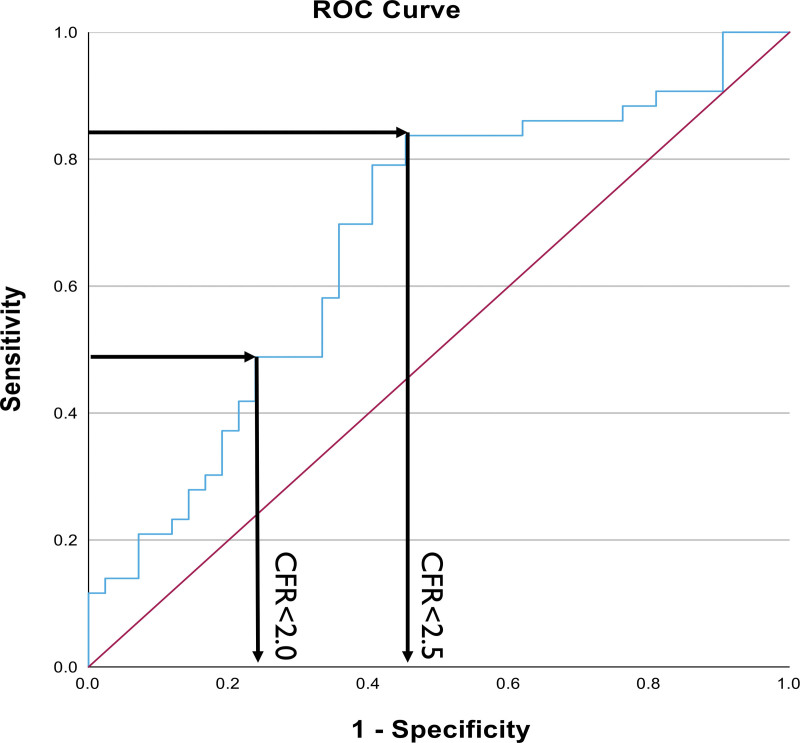
**Receiver operating characteristic curve to determine the optimal CFR threshold to predict an increment in exercise time ≥60 s in response to anti-ischemic therapy.** CFR indicates coronary flow reserve; and ROC, receiver operator characteristics.

Using linear regression analysis on all randomly assigned patients, CFR (as a continuous variable) was the only independent variable that was associated with an increment in exercise time ≥60 s in response to anti-ischemic therapy; however, the *R*^2^ for this model was only 11.9% (Table 4). The optimal CFR to predict an increment in exercise time of ≥60 s in response to anti-ischemic therapy (ranolazine and amlodipine) was 2.5 (95% CI, 2.24–2.71), sensitivity 84% and specificity 55% (Figure 4). The CFR<2.5 threshold was 69% accurate at predicting an increment in exercise time of ≥60 s in response to anti-ischemic therapy, compared with the CFR<2.0 threshold, which was 61% accurate (sensitivity 49% and specificity 74%; *P*<0.001).

**Table 4.**
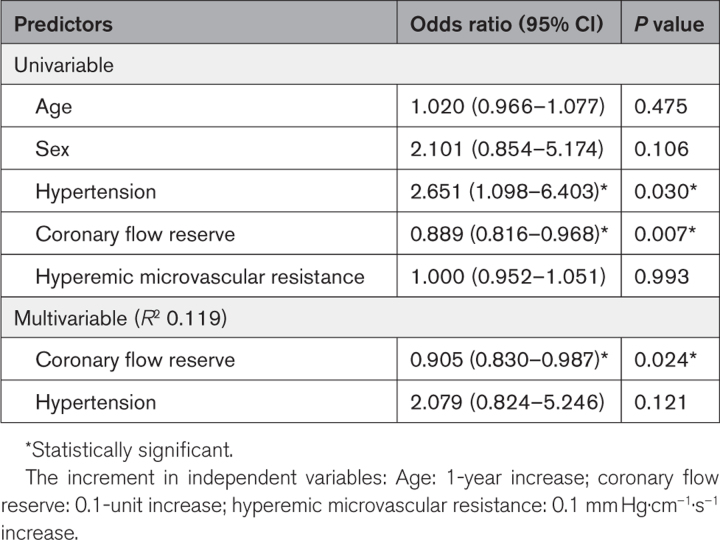
Predictors of ≥60 Second Increment in Exercise Time in Response to Anti-ischemic Therapy (Ranolazine and Amlodipine)

Patients with structural CMD had a greater numerical increment in exercise time in response to amlodipine than ranolazine (mean difference in delta, 46 s [95% CI, –2 to 93 s], *P*=0.056), but this did not reach statistical significance. Patients with functional CMD had a similar increment in exercise time in response to ranolazine and amlodipine (mean difference in delta, 3 s, *P*=0.859; Table 5 and Table S3).

**Table 5.**
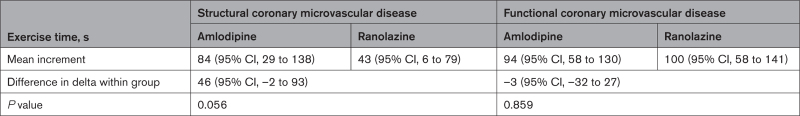
Within-Group Differences in Delta in Exercise Time in Patients With Structural and Functional Coronary Microvascular Disease.

The degree of change in SAQ summary score in response to amlodipine was similar between the CMD and reference groups (mean difference in delta, 2 points [95% CI, –5 to 8], *P*=0.549). There was a greater increment in SAQ summary score with ranolazine in the CMD group than in the reference group (mean difference in delta, 7 points [95% CI, 0–15], *P*=0.048; Table 3 and Table S1). The gain in SAQ was not lost on the cessation of study medications in the reference group; however, in the CMD group, gain-and-loss of function of SAQ summary score was observed with and without ranolazine (Figure S3).

Thirteen patients had previous exposure to amlodipine, whereas 2 patients had previous exposure to ranolazine. There was no evidence of an interaction between previous exposure to medication and the change in exercise time. Six patients (46%) with versus 23 patients (38%) without previous exposure to amlodipine had ≥60 s increment in exercise time in response to amlodipine (*P*=0.542). None of the patients with previous exposure to ranolazine had ≥60 s increment in exercise time in response to ranolazine. There was no evidence of an interaction between allocated treatment sequence and the change in exercise time. Patients allocated to the ranolazine-amlodipine sequence (n=40) had a similar change in exercise time to those allocated to the amlodipine-ranolazine sequence (n=47), in response to amlodipine (mean difference in delta, 29 s [95% CI, –19 to 76 s], *P*=0.232) and ranolazine (mean difference in delta, 4 s [95% CI, –44 to 51 s], *P*=0.877).

## DISCUSSION

In ChaMP-CMD, patients with ANOCA and an impaired CFR had an improvement in exercise time in response to anti-ischemic therapy, whereas those with a normal CFR did not. The gain in function with therapy was lost on cessation of therapy in patients with CMD, which suggests a causal link between the pathophysiological classification and response to therapy. We believe that this is the first robust demonstration of the association between CFR and an improvement in exercise capacity in response to anti-ischemic therapy. This, in turn, demonstrates the practical utility of measuring CFR in patients with ANOCA as a means of personalizing therapies. Among patients with CMD, functional CMD was the most prevalent endotype and these patients had an increment in exercise time in response to anti-ischemic therapy similar to patients with structural CMD.

We had previously demonstrated that an impaired CFR reliably identifies those with an ischemic substrate and maladaptive physiology during exercise,^2^ but whether this classification also identifies patients who derive objective benefit in their exercise capacity from anti-ischemic therapy was not known. Three contemporary trials designed to assess response to therapy in patients with ANOCA have yielded equivocal results^11–13^ and have been limited by inclusion of patients with atypical symptoms, low prevalence of impaired CFR at baseline, and the use of subjective quality-of-life questionnaires as the primary outcome.^11–13^ Post hoc analyses of two of these studies have suggested that those with an impaired CFR at baseline may derive an improvement in their quality of life in response to anti-ischemic therapy.^11,13^ The CorMicA study also demonstrated superiority of physiology-guided management above empirical therapy in relation to SAQ scores at 6 and 12 months,^7,14^ and improvement in resource consumption, as well.^15^ However, the definition of vasomotor abnormalities was broad in CorMicA (diminished CFR or elevated minimal microvascular resistance or epicardial/microvascular spasm); hence, it was not possible to link the specific pathophysiological diagnosis with outcome. Furthermore, the recommended therapies included a range of pharmacological and nonpharmacological measures (including referral to cardiac rehabilitation).^15^ Therefore, the mechanistic link between physiological findings, therapies instituted, and improvement in outcome remained incompletely understood. CFR was the only biologically plausible variable that was associated with an increment in exercise time ≥60 s in response to anti-ischemic therapy in our study. However, the multivariable model which included CFR, sex, and hypertension only accounted for 11.9% of the change in exercise time. This suggests that there must be other patient-related factors that affect the response to therapy that were not characterized in our study.

We have previously shown that patients with an impaired CFR have an ischemic substrate regardless of whether they have elevated or normal minimal microvascular resistance (structural and functional CMD endotypes, respectively); these endotypes have a similar core phenotype but disparate underlying pathobiological mechanisms.^2,6^ Other groups have since confirmed these findings in the catheter laboratory^16^ and have reported a similar adverse prognosis for both endotypes.^17^ Our finding, that patients with functional CMD derive as much benefit with anti-ischemic therapy as those with structural CMD, provides further evidence for the veracity of this endotype.

When objectively assessing the efficacy of therapies on exercise capacity, the minimum clinically relevant increment in exercise time is often regarded to be 30 s. It is notable that patients with CMD had an increment in exercise time after anti-ischemic therapy that was, on average, greater than twice this minimum difference. This treatment effect is in keeping with those reported in seminal anti-ischemic trials of patients with obstructive coronary artery disease (CAD)^18–21^ and greater than the effect reported in many trials of patients with heart failure ^22,23^ (Table S4). This therapeutic effect, and our observation that the diagnostic and therapeutic thresholds of CFR are identical, suggest a mechanistic link between the presence of an ischemic substrate and exercise capacity. We have demonstrated that the CFR<2.5 threshold can predict a clinically relevant therapeutic response with greater accuracy than the CFR<2.0 threshold. This follows from our previous study, which demonstrated that the CFR<2.5 threshold had a better accuracy at predicting myocardial ischemia and coronary perfusion efficiency during exercise than the CFR<2.0 threshold.^2^ This reaffirms a strong mechanistic link between coronary perfusion inefficiency during exercise, myocardial ischemia, and response to therapy.

The patients enrolled in ChaMP-CMD reported a degree of impairment in their quality of life, on account of angina, that was comparable to that reported by patients with obstructive CAD in ORBITA (Objective Randomized Blinded Investigation With Optimal Medical Therapy of Angioplasty in Stable Angina)^24^ and ANOCA in CorMicA,^7^ but worse than patients with obstructive CAD in ISCHEMIA (International Study of Comparative Health Effectiveness With Medical and Invasive Approaches)^25^ and ANOCA in CIAO-ISCHEMIA (Changes in Ischemia and Angina Over 1 Year Among ISCHEMIA Trial Screen Failures With no Obstructive CAD on Coronary CT Angiography)^26^ (Table S5). The minimum clinically meaningful change in the SAQ summary score, after an investigational therapy, has previously been regarded to be a difference of 8 points. By this metric, patients with CMD experienced an improvement in their SAQ summary score compared with the reference group in response to ranolazine but not amlodipine, whereas no meaningful improvement in SAQ summary score was observed within the reference group.

The discordance in treatment effect, as assessed by exercise time versus SAQ summary score, may be because the subjective assessment of quality of life is multifactorial (the coefficient of determination, *r*^2^, between SAQ and exercise time at baseline was only 15%) and more prone to treatment bias than objective measures like exercise time. The uncoupling of ischemia and SAQ scores was also seen in the CIAO-ISCHEMIA study, where some patients reported improvement in their SAQ scores after medical treatment despite having persistence of objective ischemia.^26^ Likewise, in a study of patients with obstructive CAD, percutaneous coronary intervention led to a reduction in the ischemic burden, but this did not translate to a better angina-specific quality of life.^27^ Our finding of a weak correlation between baseline exercise time and SAQ score is corroborated by a near-identical correlation reported by Spertus et al^10^ in the early SAQ validation studies. Other groups have demonstrated a weak correlation between exercise time and quality-of-life measures in heart failure with preserved ejection fraction.^28^ It has been hypothesized that exercise time and quality-of-life questionnaires measure different aspects of patients’ health and should be used together rather than in isolation.^28^

There was some evidence of a training effect, with participants walking for 22 s longer, on average, during their fourth ETT compared with the first one. Although this may have led to slight overestimation of the treatment effects overall, there is no evidence that this has affected the comparison of treatment effect between groups or between drugs, given the randomized crossover design. Likewise, patients scored 5 points higher on their fourth SAQ than on the first one; however, this did not reach the minimum clinically relevant threshold of 8 points.

There is currently marked heterogeneity in the diagnosis and management of patients with ANOCA for a variety of reasons, some of which we have addressed with the findings of our study. First, there is a widely held belief that empirical management of patients with ANOCA (without embarking on definitive diagnostic testing to confirm or refute the presence of CMD) is an acceptable alternative. We have demonstrated that patients with ANOCA, but no CMD, do not experience any improvement in objective exercise capacity or subjective quality of life with anti-ischemic therapies. To treat these patients empirically would not only potentially expose them to ineffective therapies but also delay the pursuit of alternative causes for their symptoms. The cause of chest pain in patients with ANOCA and normal CFR is unclear, but our findings suggest that inducible ischemia may not be an appropriate treatment target in this group.

We chose to study ranolazine and amlodipine as the exemplar drugs in our study, because these were expected to address the pathobiological processes that are believed to underlie each CMD endotype. The hallmark of functional CMD is submaximal vasodilatation at rest, which could be due to heightened myocardial oxygen demand at rest (although other reasons have been postulated). Ranolazine has been shown to modulate myocyte metabolism (by switching fatty acid oxidation to glucose oxidation^29^) which could decrease myocardial oxygen demand at rest and so we hypothesized that it would be most effective in patients with functional CMD. On the other hand, structural CMD is characterized by failure of vasodilatation during exercise in both the myocardial and systemic vasculature.^6^ This makes amlodipine an attractive therapy because it reduces peripheral vascular resistance, leading to reduced myocardial oxygen demand during stress, and may also cause dilatation of coronary arterioles, augmenting supply. Although patients with the functional endotype responded equally well to both anti-ischemic agents, those with the structural endotype responded better to amlodipine. These results suggest that endotype-directed therapy may be advantageous; however, further research needs to be performed to confirm these findings in a larger cohort of patients. We should also note that our sample size was designed to demonstrate a difference in exercise time of at least 60 s between each drug-endotype interaction, and so we cannot exclude smaller but clinically relevant differences between endotypes.

Our study has some limitations that should be considered when interpreting the findings. First, this was a single-center study with a relatively small sample size with inherent limitations of generalizability. Second, we did not include a placebo therapy arm, and, hence, this was not double blinded in the conventional sense. Knowledge of the medications patients were taking may have led to some treatment bias, but this would be expected to occur in both CMD and reference groups equally. Furthermore, patients and researchers were blinded to the physiological classification (phenotype-blinding) and had no means of knowing whether a given drug was expected to affect their exercise time or quality of life. Third, we did not repeat coronary physiology measurements, which may have provided an important mechanistic link. However, repeating these measurements 3 times within a course of 12 weeks would have been highly burdensome for our patients and beyond the study resources. Fourth, we have studied 2 specific anti-ischemic agents with different mechanistic profiles and therefore cannot be certain that the differential effect we observed (in CMD versus reference patients) would be replicated with other classes of drugs. Last, it is important to emphasize that this was a carefully selected patient cohort with a high symptom burden and pretest probability of coronary vascular dysfunction (Figure 2); therefore, these results may not apply to patients with ANOCA who present with atypical or minimal symptoms.

In summary, we have demonstrated that an invasive diagnosis of CMD, on the basis of CFR, distinguishes patients with ANOCA who derive objective benefit from anti-ischemic therapy. The CFR threshold that is commonly used to diagnose CMD was also the most accurate in identifying patients who respond to anti-ischemic therapy. These findings support measurement of CFR in patients with ANOCA and typical limiting symptoms to not only establish a diagnosis and predict prognosis, but also to guide medical therapy. Our findings should also inform future trials of anti-ischemic therapies for CMD, which should selectively enroll patients with diminished CFR or be designed to allow comparison of patients who have ANOCA with versus without evidence of CMD.

## ARTICLE INFORMATION

### Acknowledgments

ChaMP-CMD was an investigator-led trial funded by a grant from the Medical Research Council (MR/T029390/1) and sponsored by King’s College London and Guys’ and St. Thomas’ NHS Foundation Trust. Coauthors acknowledge support from the British Heart Foundation (FS/16/49/32320, CH/1999001/11735, and RE/18/2/34213), the UK National Institute for Health Research (through the Biomedical Research Centre award to King’s College London and Guy’s and St Thomas’ Hospital), the Fondation Leducq and the NIHR Clinical Research Network (NIHR CRN). to the authors thank Dr Pavlidis, Dr Morgan, Dr Kaier, Dr Ryan, Mr Ogden, Mr Belford, Ms Dovtartaite, Mr Campbell, Dr Li KamWa, Dr Ezad, and Mr Redmond for helping with data acquisition. Most importantly, the authors would like to thank all their participants and their families for their dedication to this research. Drs Sinha, Rahman, Marber, Webb, and Perera were responsible for the conception and design of the study. Drs Sinha, Webb, Chiribiri, Douiri, Shah, Marber (independent chairperson), and Perera (principal investigator) were members of the steering committee. Drs Sinha, Rahman, and Perera drafted and revised the document, which all authors reviewed and advised on. Drs Sinha, Rahman, Demir, Douiri, Chiribiri, and Perera performed data analysis. Drs Rahman, Demir, De Silva, Clapp, Webb, and Gulati, H. Ellis, U. Dutta, and Dr Perera were responsible for data acquisition in the cardiac catheter laboratory. Dr Douiri was the study statistician.

### Sources of Funding

The trial was funded by a grant from the Medical Research Council (MR/T029390/1). The funders of the study peer reviewed the study design but had no role in data collection, data analysis, data interpretation, or writing of the report. The first, corresponding, and last authors had full access to all the data in the study and had final responsibility for the decision to submit for publication.

### Disclosures

None.

### Supplemental Material

Tables S1–S5

Figures S1–S3

## Supplementary Material
