# Supplementary material for: ChaMP-CMD: A Phenotype-Blinded, Randomized Controlled, Cross-Over Trial
Source: Circulation. 2023 Oct 31;149(1):36–47. doi: 10.1161/CIRCULATIONAHA.123.066680 (PMC10752262; doi:10.1161/CIRCULATIONAHA.123.066680)
Supplement: Supplementary file 1 [file cir-149-36-s001.pdf]

Supplemental material

Contents

Table S1. Primary (difference in delta in exercise time) and secondary (difference in delta in SAQ summary score) outcomes (intention-to-treat analysis). 2

Table S2. Exercise treadmill test parameters 3

Table S3. Within group differences in change in exercise time in patients with structural and functional CMD (intention-to-treat analysis) 4

Table S4. Comparison of previous exemplar cardiovascular trials using exercise time as an outcome measure. 5

Table S5. Comparison of SAQ scores in other trials of patients with ischemic heart disease. 7

Figure S1. A comparison of exercise time between first and fourth ETTs. 8

Figure S2. A comparison of average daily step counts in the four-week period preceding ETTs. 9

Figure S3. Change in SAQ summary score between baseline, with anti-ischemic medication and without anti-ischemic medication. 10

Table S1. Primary (difference in delta in exercise time) and secondary (difference in delta in SAQ summary score) outcomes (intention-to-treat analysis).

	**Amlodipine**		**Ranolazine**	
	**CMD**	**Reference**	**CMD**	**Reference**
**Exercise time (seconds)**				
Increment	89 (95% CI 69 to 117)	13 (95% CI -14 to 40)	80 (95% CI 53 to 108)	5 (95% CI -27 to 37)
**Difference in delta between groups**	76 (95% CI 34 to 118)		75 (95% CI 31 to 120)	
P value	<0.001		0.001	
**SAQ summary score**				
Increment	7 (95% CI 3 to 11)	6 (95% CI 2 to 10)	11 (95% CI 6 to 16)	7 (95% CI 2 to 12)
**Difference in delta between groups**	1 (95% CI -5 to 7)		4 (95% CI -3 to 12)	
P value	0.768		0.254	

CMD: coronary microvascular disease; SAQ: Seattle Angina Questionnaire

Table S2. Exercise treadmill test parameters.

/	Baseline			Amlodipine			Ranolazine		
	**CMD**	**Reference**	**P value**	**CMD**	**Reference**	**P value**	**CMD**	**Reference**	**P value**
**Frequency of angina, n (%)**	44 (80)	23 (77)	0.710	30 (61)	15 (58)	0.766	25 (49)	14 (58)	0.451
**Reason for stopping ETT, n (%)**			0.459			0.198			0.433
Angina	29 (53)	17 (57)		16 (33)	8 (31)		16 (31)	12 (52)	
Dyspnea	15 (27)	4 (13)		7 (15)	8 (31)		17 (33)	7 (30)	
Fatigue	10 (18)	8 (27)		18 (38)	10 (38)		14 (28)	3 (13)	
Presyncope	1 (2)	1 (3)		3 (6)	0		3 (6)	1 (4)	
Musculoskeletal pain	0	0		4 (8)	0		1 (2)	0	
**Time to angina, seconds**	205±98	187±111	0.490	297±161	251±146	0.362	260±122	183±88	0.028

CMD: coronary microvascular disease; ETT: exercise treadmill test

Table S3. Within group differences in change in exercise time in patients with structural and functional CMD (intention-to-treat analysis).

	**Structural CMD**		**Functional CMD**	
	**AML**	**RNL**	**AML**	**RNL**
**Exercise time (seconds)**				
Mean increment	74 (95% CI 25 to 124)	42 (95% CI 8 to 76)	97 (95% CI 62 to 132)	101 (95% CI 63 to 139)
**Difference in delta within group**	39 (95% CI -1 to 79)		-2 (95% CI -29 to 25)	
P value	0.058		0.865	

CMD: coronary microvascular disease; AML: amlodipine; RNL: ranolazine

Table S4. Comparison of previous exemplar cardiovascular trials using exercise time as an outcome measure.

	**Study design**	**Baseline ET**	**Increment in ET**
**Obstructive CAD**			
Fox KM et al[18]<br>TIBET	Design: Double-blind parallel-group study<br>Patient numbers: 319<br>Follow-up: Six weeks	417s (atenolol arm)<br>423s (nifedipine arm)<br>410 (combination arm)	91s (atenolol arm)<br>91s (nifedipine arm)<br>98s (combination arm)
Frischman WH et al[19]	Design: Double-blind, placebo controlled, RCT<br>Patient numbers: 551<br>Follow-up: Four weeks	348s (verapamil)<br>360s (amlodipine)<br>348s (amlodipine and atenolol)<br>354s (placebo)	66s (verapamil)<br>60s (amlodipine)<br>72s (amlodipine and atenolol)<br>24s (placebo)
Chaitman BR et al[20]<br>MARISA	Design: Double blinded placebo controlled RCT;<br>Patient numbers: 191<br>Follow-up: One week of therapy at 3 sequentially doses	Baseline exercise times not reported	116s (Ranolazine 750mg)<br>70s (placebo)
Noman A et al[21]	Design: Double-blind, placebo-controlled, crossover RCT<br>Patient numbers: 65<br>Follow-up: Six weeks	301s	6s (placebo)<br>93s (allopurinol)

Al-Lamee R et al[24] **ORBITA**	Design: Double-blinded sham procedure trial; Patient numbers: 230 Follow-up: Six weeks	528s (PCI group) 490s (sham group)	28s (PCI group) 12s (sham group)
Reynolds HR et al[26] **ISCHEMIA-CIAO**	Design: observational substudy Patient numbers: 116 Follow-up: One year	364s	46s
**Heart failure with reduced ejection fraction**			
Rouleau JL et al[22] **IMPRESS**	Design: Double-blinded parallel RCT Patient numbers: 573 Follow-up: 12 weeks	511s (omapatrilat arm) 500s (lisinopril arm)	24s (omapatrilat arm) 31s (lisinopril arm)
**Australia/New Zealand Heart Failure Research Collaborative Group**[23]	Design: Double-blind, placebo-controlled RCT Patient numbers: 415 Follow-up: One year	630s	7s 24s improvement) and placebo (roughly 17s improvement)

CAD: coronary artery disease; ET: exercise time

Table S5. Comparison of SAQ scores in other trials of patients with ischemic heart disease.

	**SAQ scores at baseline**	**SAQ scores with treatment**
CorMicA[7]	Summary score 51<br>Angina frequency score 59	Summary score 52 (control arm)<br>Summary score 68 (intervention arm)<br>Angina frequency score 56 (control arm)<br>Angina frequency score 75 (intervention arm)<br>Follow-up: 6 months
ORBITA[24]	Angina frequency score 63 (PCI group)<br>Angina frequency score 60 (sham group)	Angina frequency score 74 (PCI group)<br>Angina frequency score 68 (sham group)<br>Follow-up: 6 weeks
Spertus JA et al[25]<br>ISCHEMIA	Summary score 73 (invasive arm)<br>Summary score 75 (conservative arm)<br>Angina frequency score 81 (invasive arm)<br>Angina frequency score 82 (conservative arm)	Summary score 89 (invasive arm)<br>Summary score 83 (conservative arm)<br>Follow-up: 3 years
ISCHEMIA-CIAO[26]	Summary score 83<br>Angina frequency score 90	Summary score 92<br>Angina frequency score 100<br>Follow-up: 6 months

Figure S1. A comparison of exercise time between first and fourth ETTs.

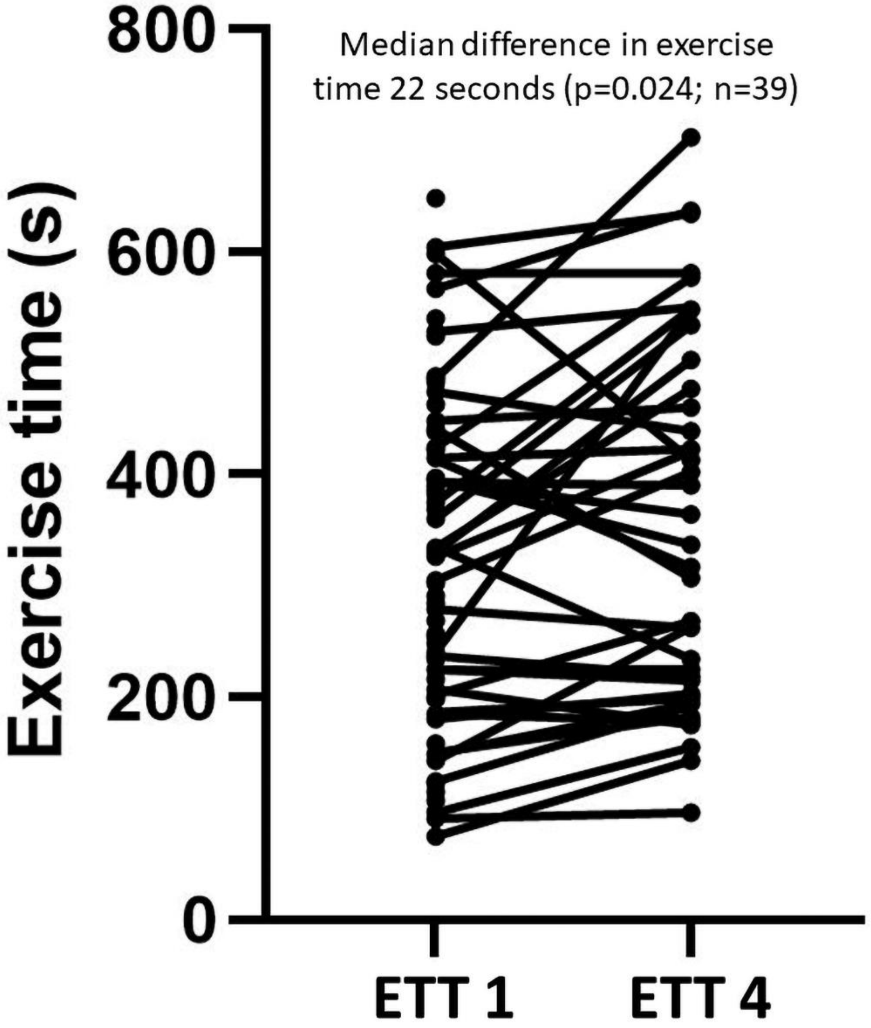

ETT: exercise treadmill test

Data are presented as individual plots; p value is calculated using the Wilcoxon matched pairs signed rank test.

Figure S2. A comparison of average daily step counts in the four-week period preceding ETTs.

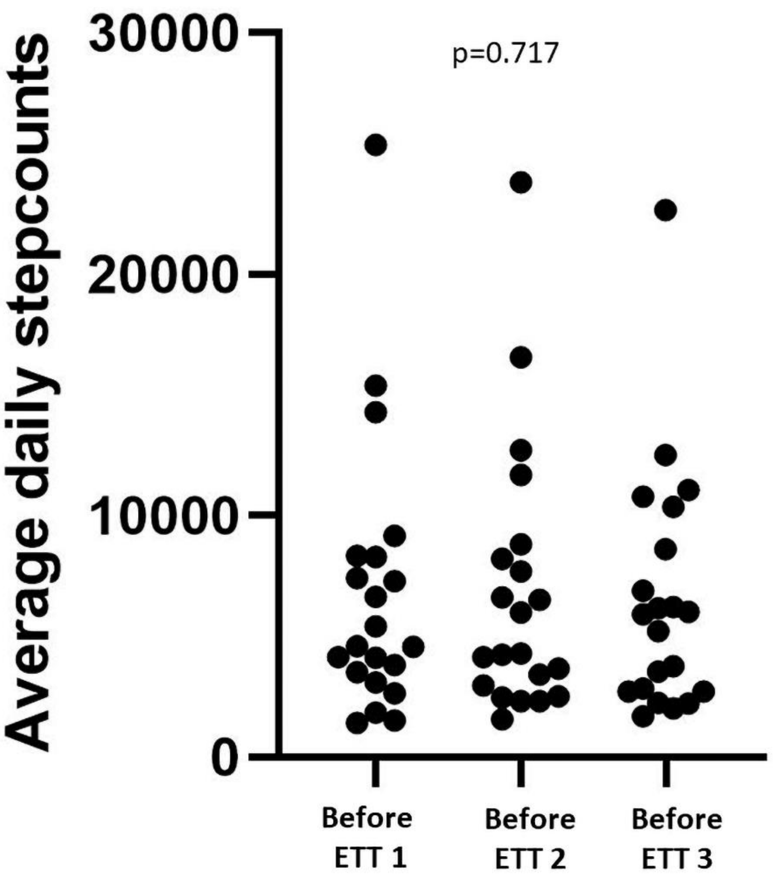

ETT: exercise treadmill test

Data are presented as individual plots; p value is calculated using the Friedman test.

Figure S3. Change in SAQ summary score between baseline, with anti-ischemic medication and without anti-ischemic medication.

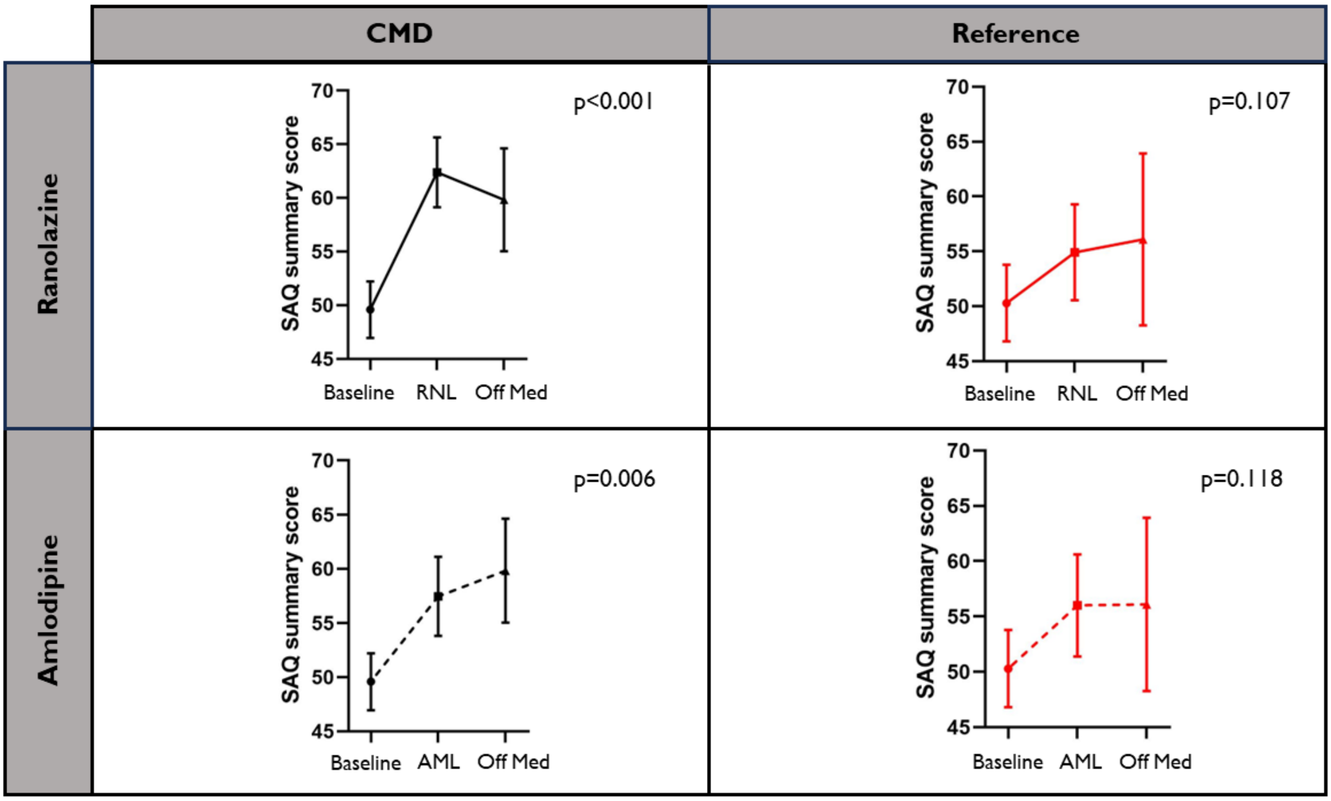

CMD: coronary microvascular disease; RNL: ranolazine; AML: amlodipine; Off Med: after cessation of study medications

Data are presented as mean±SEM; p values are for repeated measures ANOVA
